# “Care or control?”: a qualitative study of staff experiences with outpatient commitment orders

**DOI:** 10.1007/s00127-016-1193-8

**Published:** 2016-02-12

**Authors:** Bjørn Stensrud, Georg Høyer, Gro Beston, Arild Granerud, Anne Signe Landheim

**Affiliations:** Institute of Community Medicine, Faculty of Health Sciences, University of Tromsø, Tromsø, Norway; Norwegian Research Network On Coercion in Mental Healthcare and Institute of Community Medicine, Faculty of Health Sciences, University of Tromsø, Tromsø, Norway; Innlandet Hospital Trust, P.O. Box 104, 2381 Brumunddal, Norway; Faculty of Public Health, Hedmark University College, Elverum, Norway; Norwegian National Advisory Unit On Concurrent Substance Abuse and Mental Health Disorders, Innlandet Hospital Trust, Brumunddal, Norway; SERAF, Norwegian Centre for Addiction Research, University of Oslo, Oslo, Norway

**Keywords:** Coercion, Insight, Mental health professionals, Outpatient commitment, Psychosis

## Abstract

**Purpose:**

Outpatient commitment orders are being increasingly used in many countries to ensure follow-up care of people with psychotic disorders after discharge from hospital. Several studies have examined outpatient commitment in relation to use of health care services, but there have been fewer studies of health professionals’ experiences with the scheme. The purpose of this study was to examine health professionals’ experiences with patients subject to outpatient commitment.

**Methods:**

This was a focus group study using a descriptive and exploratory approach. The study was based on three focus group interviews with a total of 22 participants. Data were analysed using qualitative content analysis.

**Results:**

The study showed that health professionals had a positive attitude towards outpatient commitment and considered it necessary for patients with psychosis who lacked insight and did not collaborate on treatment. At the same time their attention to patients’ lack of insight could lead to a paternalistic approach more than measures to enhance patient autonomy. This challenged their therapeutic relationship with the patient.

**Conclusion:**

Health professionals found it difficult to combine control with therapeutic care, but gave greater emphasis to patients’ need for treatment and continuity of care than to their autonomy. This dilemma indicates a need to discuss whether increased attention to patients’ autonomy rather than insight into their illness would improve treatment cooperation and reduce the use of coercion.

## Introduction

Outpatient commitment orders (OC) are used by health professionals to ensure treatment continuity for people with psychotic disorders after discharge from inpatient care, and are generally intended for patients with many treatment interruptions and readmissions [1]. OC rests on an underlying understanding that some patients are unable to assess their treatment needs [2]. Several studies have examined whether the scheme affects patients’ use of health services [3]. Swartz and colleagues [4] found that OC improved treatment outcome when the decision was accompanied by increased treatment resources. Burns et al. [5] concluded that OC neither reduced the use of health care services nor improved patient outcome. Systematic literature reviews have supported this [1, 6]. Fewer studies have examined clinicians’ experiences with patients under OC. A study by Mullen et al. [7] showed that health professionals had different perceptions of whether the benefits of the scheme outweighed the disadvantages. Jobling [8] argued that instead of asking if OC works, health professionals should ask who OCs might work for, in what circumstances and why. Stroud et al. [9] found that OC provided security and structure in work with the sickest patients. Canvin et al. [10] showed that OC focused on patients’ medical adherence, and that patients, families and health professionals had different experiences of how the scheme affected other areas. Several studies have shown that health professionals were generally positive towards OC and considered it a useful scheme in clinical practice [11–14].

National laws vary with regard to coercive powers and the criteria for imposing OC [15]. Churchill et al. [1] identified two main forms of OC in use internationally. The “least restrictive” offer OC as an alternative to hospitalisation, while the “preventative” OCs are intended to avoid deterioration that could result in dangerousness. The Norwegian scheme is characterised by being least restrictive [16]. According to the Norwegian Mental Health Act the legal criteria for OC are the same as for inpatient civil commitment. OC is normally established after compulsory admission to hospital, although Norwegian legislation also allows for OC without prior hospitalisation. The only coercive intervention that can be imposed on patients on an OC order in Norway is that patients who do not attend treatment appointments can be brought from their homes, by physical force if necessary. If patients under OC refuse treatment, a separate compulsory treatment decision is needed. Rehospitalisation of patients under OC requires a simple procedure where the responsible clinician can decide to readmit the patient without the need for any new independent assessment [17].

OC in Norway can only be decided by a psychiatrist or a specialist psychologist. Depending on the patient’s location and care needs, follow-up care may be provided by health professionals from either specialist or local health services, or through coordination between the two. OC is monitored by an independent supervisory commission (the Control Commission) which also serves as a complaints board for OC patients. The Control Commission must, on an independent basis, approve compulsory interventions longer than 1 year.

Although the use of OC is increasing, there is scant knowledge of how the scheme is practised. The present study is part of a larger study of OC in Norway which also examines patient and family experiences of OC [18–20]. The purpose of the present study was to examine health professionals’ experiences with patients subject to OC.

## Materials and methods

To answer the research question, we conducted three focus group interviews with a total of 22 participants. The study had a descriptive and explorative design using qualitative content analysis as described by Graneheim and Lundman [21]. This method focuses on the subject and the context, emphasising both similarities and differences.

### Recruitment and setting

The study was conducted in two counties in Eastern Norway. The counties consist of small towns and rural regions with a total population of 383,000. The specialist mental health services in the two counties consist of two hospitals and five district psychiatric centres (DPC). The DPC represents a treatment level between local authority health services and hospitals. The 48 local authorities in the area studied also provide community mental health care. A written invitation was sent to heads of department in one hospital, four DPCs and four local authorities. The other hospital had transferred all patients to a DPC. The DPCs were chosen because they provided follow-up care for the majority of patients subjected to OC, the local authorities because two had hospitals in their areas and two were towns with large DPCs. The sample was strategic, aiming to capture health professionals with experience of follow-up care for OC patients. Recruitment was aimed at clinicians responsible for OC decisions and/or follow-up care, and at least one year’s experience of working with OC patients. All health professionals willing to participate in the study were included. Due to lack of time, one of the four invited local authorities did not prioritise participation in the study.

### Participants

The sample consisted of 13 women and 9 men who were divided into three focus groups (Table 1). Focus Group 1 consisted of health professionals who exclusively engaged in treatment and follow-up care of patients, but without authority to enact OC (care providers). Focus Group 2 consisted only of those authorised by law to make OC decisions (decision makers). Focus Group 3 consisted of those who were unable to attend the first two interviews, and was thus composed of care providers and decision makers. The purpose of dividing into the first and second group was to explore the participants’ experiences on the basis of different responsibilities. In addition to this, the goal of the third group was to encourage a discussion that could provide a nuanced view of cooperation between care providers and decision makers.

**Table 1:** The participants’ profession, place of work and distribution in the focus groups

	Focus group 1	Focus group 2	Focus group 3	Total	Hospital	DPC	Community mental health care
Decision makers							
Psychiatrist		4	2	6	1	5	
Psychologist			2	2		2	
Care providers							
Mental health nurse	7		2	9		5	4
Nurse			2	2	2		
Nurse assistant	1		2	3			3
Total	8	4	10	22	3	12	7

The decision makers all worked for the specialist health services (*n* = 8) and had overall responsibility for treating the patient. The care providers came partly from specialist (*n* = 7) and partly from community health services (*n* = 7), and ensured the daily follow-up care of the patient within the OC framework.

### Data collection

The focus group interviews were conducted using a thematic interview guide based on the research group’s theoretical knowledge and practical experience of OC. A staff member with user experience participated in the design of the interview guide. The interviews started with an open question asking participants to share their experiences of follow-up care of patients subject to OC. Subsequent questions differed between the groups in concentrating either on the responsibility for follow-up care or on the OC decision itself. The third focus group had the same opening theme, while the later questions probed the cooperation between decision makers and care providers. All interviews were conducted in November and December 2014, took place in a hospital setting and lasted about 2 h each. The interviews were conducted by the first author (moderator) together with a co-moderator, the staff member with user experience mentioned above. The moderator led the interviews. The co-moderator listened and noted down thoughts that arose when following the dialogue. The co-moderator was invited to join in to share her reflections and ask for more detail. The interviews were recorded and transcribed verbatim.

### Analysis

The analysis was performed in steps [21] where each interview was read through several times, and then subdivided by identifying meaning units in the transcribed text. A meaning unit is a constellation of words or statements that relate to the same central meaning [21]. The participants’ experiences working with patients on OC orders were emphasised [22]. The next step was shortening the text while preserving the core of the meaning units. This condensed text was labelled as an indicator, covering the intended meaning. The third step was to sort the indicators into subcategories and categories. Three categories were identified: responsibility and OC, therapeutic alliances and OC and difficult decisions and OC. The categories were descriptive and understood as expressions of the manifest content in the text. The final step was interpretative, creating a theme linking underlying meaning from the indicators, subcategories and categories to achieve a new level of understanding. The main theme of care or control was understood as an expression of the latent content of the text [23]. The analysis was carried out by the first author. In working with the data material, the moderator and co-moderator met several times to discuss the development of sub-categories, categories and a main theme. The analytical steps and interpretations were also discussed in the research group to validate the understanding. The context and analytical steps are outlined to enhance trustworthiness [22]. Subcategories, categories and the main theme were compared with the interview data to ensure that they covered the participants’ stories as they were told. Sorting the interview data into categories was supported by using NVivo 10 (Alfasoft, Sweden).

### Ethical considerations

The participating health professionals were informed verbally and in writing about the study before signing a written consent form. They were told that participation was voluntary and that they could withdraw consent at any time without any consequences. All data were kept confidential and stored in a de-identified form. No names are used in the presentation. The study was approved by the Data Protection Officer for the health region where the study took place.

## Results

### Care or control

The main topic recurring in all focus groups was the problems experienced by clinicians in balancing the provision of help and care with managing coercion. This overarching finding is presented through the three categories that emerged through the content analysis of the material.

### Responsibility and OC

Participants experienced responsibility in OC as a social responsibility they managed as health workers. They had a common understanding of which patients should be subjected to OC, i.e. mainly those without sufficient insight to assess their own treatment:

Most people have a history where you can look back and see they got worse after they stopped taking their medication. They all have a history where voluntary cooperation has been tried. So there’s been a discontinuation of medication and deterioration. So it’s a way to ensure they get the treatment they need. (Psychologist).

All participants believed that stable medication would improve patient functioning, but would not always improve the patient’s cooperation on medication. One mental health nurse elaborated on her views on the consequences of a lack of insight:

Patients under OC need the same as other patients. The need is the same; it’s their lack of insight into the illness that makes them make unfortunate choices for themselves. It means that they need a framework where they cannot opt out of treatment. And they have a legal right to be taken care of.

The participants felt that OC safeguarded the interests of patients by preventing adverse events:

I have a lady who has lots of experience of stopping taking medication. She becomes manic. Then there’s a long period of time before she comes for treatment, and that gives her a chance let herself go in many arenas. Then she takes a long time to recover. So with the contact we have now [OC], we can avoid these unfortunate side effects, if one can call them that. (Mental health nurse)

The participants considered being subjected to OC as a minor intrusion in patient autonomy:

I think that in most relationships within the [OC] framework, there is plenty of room for movement. The [OC] framework is basically about medication. And attendance. Generally, the rest of the patient’s life is up to them, as far as receiving help goes. (Psychiatrist)

Participants considered OC to be a necessary measure to safeguard patients’ treatment needs when they were unable to ask for medical assistance themselves. At the same time, they found that the coercion in OC interfered little with patients’ lives. The decision maker, in cooperation with the care providers, attempted to fit patient wishes into the established treatment framework. However, decision makers were reluctant to change the OC framework also when they assessed the patient as stable. Decision makers found it particularly difficult to judge when OC could be terminated, being concerned that the patient might have a relapse.

### Therapeutic alliances and OC

The participants realised that patients might feel that OC restricted their freedom. As health workers, they nevertheless felt that the patient’s long-term health had priority over a “here and now” perspective. At the same time, OC complicated their therapeutic work:

It does something to the relationship when you’re operating with coercion. It’s important to be aware of the type of treatment you’re giving the patient. If I want to have therapeutic communication with a patient, OC is a poor starting point. (Psychiatrist)

In some places, the mixture of care and control was resolved by giving one therapist administrative responsibility for the OC, while another was in charge of the treatment. Such a solution was not possible in all locations due to lack of staff resources.

Relationships with patients were felt by the participants to be good in most areas. When it was difficult to establish cooperation, this often originated in a disagreement on medication. However, the psychiatrists in particular thought that OC assured medication because the same psychiatrist was in charge whether the patient was in the community or an inpatient. An experience common to psychiatrists was that many inexperienced doctors had put the patient on adverse drug regimens with an increased risk of side effects, before OC commenced. One psychiatrist stated that OC provided better continuity. If the patient related to a single psychiatrist, it was easier to work towards a common understanding.

Care providers experienced being a link between the decision maker and the patient. They ensured the medication was taken and had a dialogue with the patient and decision maker about treatment efficacy and side effects. At the same time, they tried to downplay the coercive framework and worked instead within a framework of milieu therapy. They were most interested in the patient’s everyday life within the OC framework:

We try to get the patient to accept OC as a measure, and then put it aside. And instead discuss with the patient—what might be useful? It’s one thing being subject to OC. But then there’s the other part, what might be useful in the opportunities provided by OC? I have argued a lot around the matter of safety. It’s a kind of safety net that allows you to be more easily checked and prevents you from becoming as ill as we know you can be. (Mental health nurse)

Care providers said that OC gave the patient the chance to stay in the community. They channelled any conflicts about medication to the decision maker to allow themselves to focus on the patient’s abilities and coping.

Most participants experienced a good flow of information about OC between health professionals, but found that relatives sometimes received insufficient information. Some participants were pragmatic and gave relatives information that they believed to be in the patient’s best interest. Others found confidentiality to be an obstacle to cooperation. Several participants said that patients had asked staff not to give them information that reminded them of negative past experiences. On other occasions, clinicians limited information because they felt it could be detrimental to cooperation with the patient.

### Difficult decisions and OC

Participants were responsible for implementing OC, but found it challenging to decide on coercive interventions when patients were opposed to medical help:

It’s an ethical dilemma. Whenever we decide, OC or no OC. Should we treat them? We’ve got patients who are really quite sick. But they do live their lives and bumble about in their world. Without bothering the community. Then we have a group where we use a risk criterion. Because if someone is dangerous, you’ve got to do something. But in other cases, we really have to think. Can we do something else to help them get their lives in order without forcing them? (Psychiatrist)

There was broad agreement on the necessity of OC when it was justified by a risk of danger to the patient or others. But it was more difficult in cases where OC was justified by a treatment need. Having to make assessments affected the participants’ lives:

I feel this responsibility entails some stress. You’re intervening and deciding something which the patient may experience as coercion, though he may later see it as representing cooperation. So I find having this responsibility affects my stomach a bit sometimes. It’s not easy to make these assessments. Knowing when you can test the alliance by ending the coercion or not. That’s the unpleasant thing about this role. Trying to make good assessments for the good of the patient. (Psychologist)

The participants found it challenging to balance the patient’s resistance with what they believed were the patient’s treatment needs. A dilemma especially highlighted by decision makers was the question of using OC if they considered that the treatment had little effect:

One of the hardest things for me to judge is the expected effect of the treatment. And relate this to the use of coercion. What if you get a partial effect or a small effect from the coercion? What’s the limit for justifying the use of coercion? And can we justify the use of coercion for years when there’s no great effect? I find that a difficult dilemma. Because coercion is abuse if it has limited effect. (Psychiatrist)

Some decision makers experienced pressure from other healthcare professionals regarding more extensive use of coercion within the OC framework:

Perhaps some local health workers expect OC to imply that you can force people into activities. Or to stop drinking or something like that. Controlling their drinking. (Psychologist)

However, participants from hospitals and DPCs thought that such pressure had diminished in recent years because local care workers had learned more about follow-up care for OC patients.

Use of physical coercion was rare; the participants’ experience was that patients complied with OC even if they did not consent or protested verbally or physically. Participants emphasised the importance of evaluating OC against the alternatives:

I don’t think we have too much coercion, I’m more concerned about using it in the right way. I’m sceptical to statements about there being too much coercion. I think we need to look at how it’s used. I wonder whether coercion should be used more in some contexts. There are always people who can’t accept what would be medically beneficial, they ought to have medication. You might find that if they’d had this intervention, they’d have avoided new psychoses. (Mental health nurse)

All participants found that the patient population under OC had changed. Previously, these patients had considerable experience of mental health care. Now there were younger patients with less experience. Decision makers felt that young patients meant greater aspirations for treatment:

We’ve got younger patients, we have to cure them. Before we had to improve patients so they felt ok. Now we have to cure them. We’ve started putting young people on OC. That’s a quite different responsibility. They should get out of OC as soon as possible, they should get off the troublesome medications as soon as possible, they should have a plan to get off medicines completely. (Psychiatrist)

Several participants said that patients’ finances and local resource priorities were aspects they included when considering OC. OC provided free medicines, and participants were unsure whether patients would prioritise medicines if they had to pay for them. Some participants saw a pattern where OC was necessary to ensure local follow-up care for the patient. Both patient cooperation and external factors were therefore included in the OC assessments.

## Discussion

This study showed that health professionals experienced OC as a social responsibility, and justified it as a necessary approach to patients with psychosis who did not cooperate on treatment. But the participants also said that discretion played a large part in OC practice. The clinicians believed that when psychotic patients did not voluntarily comply with treatment, it was because they lacked insight into their own disorder. In a discussion of insight in schizophrenia, Dam [24] showed that insight must be understood beyond the patient’s compliance with medical treatment. It is equally a question of how well patients understand and cope with their everyday lives. Insight thus has several levels and cannot be the only explanation for non-compliance with treatment. Our findings are supported by Cairns et al. [25], who showed that health professionals often justified coercive treatment with the patient’s lack of insight, without considering the degree of coping or whether the patient could participate in any decisions. Our study participants thus had a narrow understanding of insight, emphasising the patient’s compliance with what clinicians believed was the right approach more than an assessment of the patient’s understanding of his/her own situation. The participants’ attitude to patients was more paternalistic than empowering them to take responsibility for their own lives [26].

The participants felt that the coercive framework of OC challenged their therapeutic relationship, the coercion itself becoming a burden. Other studies have demonstrated the same concerns [14, 27]. It was difficult to assess when the benefits outweighed the drawbacks, especially where clinicians assessed the treatment effect of coercion as small. Romans et al. [13] found that coercion could hinder the therapeutic alliance at first, but that the benefits of stable aftercare over time offset the short-term disadvantages. Our study showed similar findings in that health professionals related OC to their responsibility to safeguard the patient’s health in a long-term perspective. As in Dawson and Mullen [28], health professionals’ clinical experience was that OC over time stabilised and improved the patient’s situation. Reviews of the state of knowledge have not shown that OC reduces readmissions and hospital days [29]. Nor is there any scientific knowledge showing that coercion improves treatment outcomes [30]. As summarised by Norvoll [31] coercion can have a negative effect on treatment outcome.

However, clinicians believed that some patient groups needed coercion to establish an effective treatment framework. They viewed OC positively as a necessary scheme for some patients to ensure compliance with medication. A previously little mentioned finding was that OC also improved the stability of the medical treatment from health professionals. Better continuity in medication coincides with the findings of Canvin et al. [10], and is in accordance with recommendations for preventative use of medicines for psychotic disorders [32]. At the same time, studies have shown that the efficacy of antipsychotics in long term treatment may be overstated [33, 34]. Our study found greater treatment aspirations in the case of young patients, where OC was used more dynamically with the aim of a rapid reduction in medicines and coercive frameworks. This finding contrasted with other parts of the study showing a more conservative practice governed by the clinicians’ fear of patient relapses.

When patients were subject to OC, it influenced the care providers and their milieu therapy. Care providers were in an intermediate position, having influence, but no authority to change the decision. They worked closely with the patient, focusing on workable solutions in the patient’s everyday life. Lorem and Hem [35] pointed out that conflicts could arise in encounters between a medical understanding of psychosis and patient-oriented care. However, our study found little conflict between decision makers and care providers. They appeared to be in agreement, and care providers followed up the OC decision while also assisting the patients in their everyday lives. It was a paradox that patients on the one hand were considered to be without responsibility for their own treatment, while the goal of milieu therapy was to make patients responsible for their lives [36]. A danger pointed out by Weller [37] was that a comprehensive patient focus becomes less comprehensive when a strong focus on medication side-lines other treatment approaches.

An emphasis on medical treatment programmes may invalidate the patient’s opinions by understanding lack of insight as illness rather than considering the patient’s history and wishes [38]. This approach breaks with current knowledge that shows the importance of patients’ participation in their own recovery [39] and with studies that have shown improved treatment outcomes when patients use their own resources [40, 41]. The fact that our study revealed little use of physical coercion may be because the patient’s increased understanding led to an acknowledgement of the need for treatment. But it may also mean that the patient passively adapted to the clinician’s authority.

Health professionals considered OC to be a small intrusion in the patient’s autonomy. This finding contrasts with those of other studies showing that OC patients felt the presence of coercion in many aspects of their everyday lives [18, 20, 42, 43]. In cases where coercion was a burden, this was linked to the clinician-patient relationship. Different understandings of the patient’s healthcare needs can complicate the therapeutic alliance [10]. Our results showing that therapists justified OC as a means to support the patient concur with findings in other studies [11, 14, 27]. This also agrees with the fact that three out of four OC decisions in Norway are based on an assessment of the patient’s treatment needs [44]. Sjöström et al. [16] pointed out that the use of OC may downplay treatment that encourages patient participation. “Care or control” thus refers to health professionals’ dilemma in having responsibility to provide treatment while coercion at the same time could prevent patients becoming more independent.

## Strengths and limitations

The study was conducted in a limited geographical area and may have captured a local practice. Also limiting factors in the selection of participants may have been work pressures and different desire to share their experiences. However, the selection had breadth in including different treatment environments and professions. Focus groups as a data collection strategy are considered a relevant approach when the purpose is to examine peoples’ experiences, attitudes or viewpoints. However, Malterud [45] mentions the risk that focus groups may provide idealised stories, curb individual detailed experiences and exert pressure towards a consensus. We found, however, that the interviews yielded diverse experiences through good descriptions. The first author had previously worked at one of the hospitals. This provided an insider perspective that was helpful in interviews, but could also hinder academic distance [46]. The aim of the study was to use a qualitative approach to describe health professionals’ experiences of working with patients subject to OC without any intention of generalising these experiences. However, findings that concur with other studies enhance this study’s validity beyond the particular participants involved [22].

## Conclusion and clinical implications

The main finding was that health professionals found difficulty in balancing the role of therapist with the management of coercion. Health professionals had a positive view of OC, believing it was necessary to safeguard the patient’s health in a long-term perspective. They justified OC with the patient’s lack of insight to assess his/her own treatment needs. Health professionals judged that OC limited patients’ autonomy to a minor extent and felt they had a good relationship with patients. However, attention to the patient’s lack of insight led to a paternalistic approach more than measures to enhance patient autonomy. There was general consensus on roles and responsibilities in OC between the clinicians involved. But they found the management of coercion to be burdensome in that OC challenged their therapeutic relationship and treatment ideology. Increased attention to OC patients’ perceived lack of autonomy rather than their assumed lack of insight into their illness could improve treatment cooperation and reduce the use of coercion.
